# Notes and Notices

**Published:** 1890-07

**Authors:** 


### NOTES AND NOTICES.

Actinomycosis.—This is the name of a new disease which is spreading among cattle. It is popularly known as "Cancer-jaw," "big-jaw," "lump-jaw," and "lumpy-jaw." The scientific name, actinomycosis (or actinomykosis, as it is sometimes printed), is derived from the vegetable organization called actinomyces, one of the fungi, or mold, so named by Harz, professor of botany at Munich, about twelve years ago, the word coming from two Greek words meaning ray—the microscopic appearance of the organization being that of an eccentric radiating structure—and fungus. The disease was first thought to be a form of consumption, or identical with it, but it is not, nor is it necessarily confined to the jaws or its bones, but may invade the mouth, tongue, nose, stomach, lungs, udder, or skin. In many instances has the fact that the disease is inoculable been proven, and hence it is considered transmissible and infectious under favorable circumstances, especially when an injury resulting in an external or internal wound has been received.

Mr. George Fleming, of England, one of the few men to thoroughly investigate this disease said: "The progress of pathological research is continually demonstrating the mighty part played by microscopic vegetable organisms in the production of disease in plants and animals, generally leading to their destruction, and with more or less rapidity. The feeblest and smallest as well as the largest and most powerful are alike exposed to the ravages of these invading relentless foes, whose attack is all the more destructive because it can rarely be detected at the onset; and their extreme minuteness and tenuity, as well as their insidious and obscure manner of operating are also so many barriers to timely recognition and protective measures against their assaults."

He thinks it probable that the animal contracts the disease from eating mouldy hay or straw (in other words, food with the fungus already on it), especially if there are scratches, fissures, abrasions, or wounds in the mouth or on the jaw, though he does not say that it may not occur in some other way. In animals the tendency is to cause new-formation tumors, and hardening or degenerations of tissues, but in man (and he records sixteen cases in man), it tends to cause suppurative processes and metastatic abscesses. Until within a few years many cattle diseases, so-called, were known, which are now known to be only different manifestations of this one disease, modified by location principally, so I will now describe a typical case, as affecting the typical point—the jaw—as described by Mr. Fleming:

In 1877 Professor Bollinger, of Vienna, drew attention to this disease, which he claimed was frequent, which consisted in a kind of new-formation tumor that appeared on the upper or lower jaw, at the roots of the teeth, or sprang from the spongy tissues of the bone, gradually displacing the teeth, invading and destroying the healthy tissues, muscles, and skin, abscesses forming, the tumor at times reaching the size of a child's head, the bones being reduced to an appearance resembling pumice stone. (He found another form affecting the tongue, and within a year had six tongue specimens sent him from Bavaria alone.) I need not detail the minutiæ pertaining to the microscopical appearances found, or how they were arrived at, and will simply say that the actinomycosis fungus or mold is, in many respects, similar to the common green mold, pencillium glaucum, which grows on paste, jam, damp leather, etc. It is enough to say that the affected animal would soon show emaciation and debility, resulting largely from its inability to masticate its food, and death would result; but this result rarely follows, because the course of the disease is ended more summarily—by death at the hands of the butcher in order to turn the animal into "beef." A frequent complication in one of these cases is the inevitable one that as soon as a tumor gets opened on the outside it becomes fly-blown, and consequently maggotty, so that the poor animal writhes with the tortune of having to entertain a convocation of politic and vigorous worms who lunch off his own flesh.—*Dr. Wm. B. Clarke, in the Indianapolis Journal.*

Removals.—Dr. A. B. Eadie has removed from 237 King Street to 137 Church Street, Toronto, Canada. Dr. C. S. Durand, from Mungelo to Hurda, Central Provinces, India. Dr. Charles H. Young, from Baltimore to 1248 Bedford Avenue, Brooklyn. Dr. C. Eurich, from 80 Second Avenue to 119 East 86th Street, New York City. Dr. Wm. R. Powel, from 3718 Chestnut Street to 3735 Walnut Street, Philadelphia. Dr. F. C. Hood, from Marysville to Alta, California. Dr. H. H. Crippen, from San Diego, California, to 78 Maiden Lane, New York, where he will take editorial charge of *The Homœopathic Journal of Obstetrics*, published by A. L. Chatterton & Co. Dr. L. A. Ren Dell Goodrich, from Hartford to Sayville, New York. Dr. F. W. Grundmann, from 2344 Washington Street to corner Jefferson Avenue and Wash Street, St. Louis. Dr. E. T. Balch, from South Bend, Washington, to Summerland, California. Dr. D. Albert Hiller, from 1011 Sutter Street to 220 Montgomery Avenue, San Francisco. Dr. O. B. Gause, formerly of Philadelphia, has established his office for the summer at 302 Asbury Avenue, Asbury Park, New Jersey. His winter office is at Aiken, South Carolina.

At the meeting of the Indiana Medical Society (allopathic), held in June last, Dr. J. L. Thompson read a paper that bore the strange title, "An Ischiophagus." It referred to the famous freak, the Tipton-county twins. This paper was by Dr. T. O. Armfield, who gave a medical description of this two-headed baby. He noted that the brains of both were well developed, and the children quite handsome and exceedingly bright for their age. One would cry while the other laughed or slept, and one would experience pain while the other suffered none. They were put upon a museum circuit, September 21st, when three months old, and died at Buffalo, N. Y., February 21st, 1890, when eight months old. One died from measles and the other died forty-five minutes thereafter, from shock caused by the cold blood rushing into its veins from the dead child, and its inability to oxygenize the dead blood. "How much longer these children might have lived had they not contracted the measles, or some accident befallen them," said the Doctor in conclusion, "we cannot tell, but it is probable they had lived their allotted time. They had arrived at an age when nature demands some exercise, and as they could neither be set up nor turned over sufficient to relieve a congested organ or a part of an organ, I am inclined to believe that some part would have soon suffered from a non-equalization of the circulation." The Doctor thought their lives had been lengthened by the constant exhibition they had been given every hour through the day, when they were seen and examined in every possible position.

THE ELECTRIC RAILWAY AS A SANITARY MEASURE.—The rapid extension of the electric street-car system which has taken place (especially in this country), naturally leads to the question of the cause thereof. To have gained such pre-eminence it must be able to do not only what other systems can do, but still more, it must be able to do it at a decreased cost. Again, removal of thousands of horses from the streets of a city, involving, as it does, the doing away with the noise and dirt, is another distinct gain to its residents. But if one goes still further, and contemplates the difference between a stable housing thousands of horses, and an electric car station of sufficient size to operate a road with the some efficiency, one is at once struck with the advantages on the side of the electric system, which, indeed, are incontrovertible. Instead of a large, ill-smelling building, whose odors are wafted for many blocks (making the tenancy of houses within half a mile almost unbearable, and involving a large depreciation of property in the neighborhood), there is a neat, substantial building, equipped with a steam plant and dynamos, and occupying hardly one-tenth the space required for an equivalent number of horses. Therefore, not only is there effected a removal of the nuisances attached to a stable, but a large saving in the cost of real estate, and the far greater amount involved in the known depreciation of the surrounding property. Besides this, the stables are of necessity required to be in close proximity to the track, whereas the electric power station, which furnishes current to the car, may be situated a mile from the track in some suitable place, as for instance, beside a river, where, with condensing engines, power may be generated at a minimum cost. —*Exchange.*

CONSUMPTION NOT CONTAGIOUS.—The annual session of the State Medical Association opened at Pittsburg, June 10th. One hundred and fifty delegates were present from all parts of the State, and a number of interesting papers were read. Among them was one by Dr. Thomas J. Mays, of Philadelphia, on the relation of artificial inoculation to pulmonary consumption. In the course of his address Dr. Mays said:

"The contagiousness of consumption is an old idea and its logical remedy, viz., isolating the sick, was thoroughly tested in Naples from 1782 to 1848. In every case the ceilings, walls, floors, doors, and windows of the rooms in which consumptives died were torn out, burned, and new ones substituted. The bedding and furniture shared the same fate, and such dwelling were not inhabitable for one year. Consumptives were regarded as pests, and their families were shunned and often driven to want. These laws brought no amelioration after existing for sixty-six years. When the resolute and vigorous though vain efforts which the Neapolitans put forth to crush out this disease are compared with the advice of our modern contagionists, the latter seem more like the vaporings of a child's brain than the outcome of thoughtful and sober judgment. Indeed, it is a sad reflection to find men at this late day who are again willing to repeat the superstitious follies and foibles of a century ago."—*Philadelphia Times.*

STATISTICS OF FARMS, HOMES, AND MORTGAGES.—In consequence of the public interest manifested in the investigation now being prosecuted by the Census Office in relation to recorded indebtedness of private individuals and corporations and the statistics of farms, homes, and mortgages, and in view of the fact that every day letters are received at the Census Office asking for information on this subject, it has been deemed advisable to print a letter, dated May 15th, from the Superintendent of Census, in reply to a Senate resolution, as a bulletin. It contains a full statement of the character of the inquiries referred to and a description of the methods adopted. Copies may be had on application to Robert P. Porter, Superintendent of Census.

SOUTHERN HOMŒOPATHIC MEDICAL COLLEGE.—As a result of a controversy between the Maryland State Homœopathic Medical Society and the Baltimore Homœopathic Free Dispensary, on North Greene Street, the Society has resolved to establish another free dispensary and also a medical college. The college was incorporated May 15th under the name of the Southern Homœopathic Medical College and Hospital of Baltimore by Dr. Elias C. Price, Dr. Henry Chandlee, Dr. Nicholas W. Kneass, Levi Z. Condon, Dr. Robert K. Kneass, Dr. Eldridge C. Price, Dr. Michael J. Buck, Dr. Robert W. Mifflin, George M. Lamb, Dr. Henry W. Webner, Dr. Oliver Edward Janney, Dr. Henry F. Garey, Dr. Edward H. Condon, Dr. Frank C. Drane, Dr. John Hood, Martin Lane, Aubrey Pearce, Henry F. Garey, Albert N. Horner, Sebastian Brown, John T. Graham, Joshua Regester, Dr. Charles H. Thomas, Wm. A. Carroll, Woodward Abrahams, and Peter Thompson. The incorporation is for the purpose of maintaining a medical, surgical, dental, pharmaceutical, and veterinary school or college and hospital. A feature of the college will be the admission annually of one white person (male or female) from each congressional district of Maryland to every course in the college, the admission to be upon the recommendation of the Representative in Congress. The capital stock is placed at $50,000, which may be increased to $200,000, divided into $25 shares. The same gentlemen also incorporated the Maryland Homœopathic Free Dispensary and Hospital of Baltimore. It has no capital stock, its funds being derived from contributions. It is stated that about a month ago the Board of Directors of the dispensary on Greene Street were approached officially by the State Society for the purpose of discussing what the relationship of the dispensary should be to the Society at large. The Dispensary Board refused to confer upon the subject, and the incorporation was decided upon. It is said that the old dispensary has been controlled by a few physicians antagonistic to the State Society, and the Society has had no hand in its management, although the public thought it had. There was a stated meeting of the Society on Wednesday evening, at which the proposed incorporation was announced and heartily indorsed. At the meeting Wednesday the resignations of Dr. Thomas Shearer, Treasurer, and Dr. D. H. Barclay were received. Dr. M. Brewer, who resigned as President at a previous meeting, was succeeded by Dr. Elias C. Price, and Dr. O. E. Janney was elected Treasurer. No specific plans have yet been made, but the Society expect to erect a handsome college building.—*Baltimore Sun.*

An Appeal from the Census Bureau.—No organizations in the United States have multiplied more rapidly in the past ten years than the sick-benefit, funeral-aid, death-benefit, and other kindred societies.

As they are generally confined to those who are in the humbler walks of life, the good they have done is incalculable, carrying substantial aid to thousands of stricken families and inspiring those who are fortunate enough in being members with a courage which might not exist in their hearts without them.

The members of these organizations will be glad to learn that Hon. Robert P. Porter, Superintendent of the Eleventh Census, will endeavor to secure the statistics of the noble work these associations are doing, and it is safe to say that no other branch of the census will be more interesting.

The business of gathering the data has been placed in charge of Mr. Charles A. Jenney, special agent of the insurance division, 58 William Street, New York City, and all associations throughout the United States, whether incorporated or private, should assist by sending to him the address of their principal officers.

Any one interested in the sick-benefit, funeral-aid, and death-beneficiary associations of the United States can help make the statistics of their organizations for the forthcoming census more complete and disseminate the knowledge of the good work they are doing by sending the names of such societies as they may know of, and the addresses of their principal officers to Mr. Charles A. Jenney, Special Agent of the Eleventh Census, 58 William Street, New York City.

The Value of Journals to the Medical Profession.—One of our subscribers thus writes to us concerning medical journals in general and this journal in particular: "I must say I have gained a great deal of encouragement from your journal. For from it I find that the lights of Homœopathy do things just as I do; and have to study just as hard; and to use *books* in prescribing for obscure cases. I am well repaid for the money expended in subscriptions to THE HOMŒOPATHIC PHYSICIAN by the encouragement received and the *very many* most excellent hints.

"The doctor who reads medical journals lives in a healthy medical atmosphere, gaining light, air and sunshine, preventing his deteriorating and retrograding into the slimy ooze of palliatives.

"I think a doctor needs encouragement as much as any other man, and he gets it through the medium of medical journals that show him the path traveled by the brighter lights of the profession."

THE BROMIDES.—It is said that a ton and a quarter of bromides are annually consumed by the patients of the National Hospital for the Paralyzed and Epileptic in London. Poor Epileptics! Helpless Paralytics!

> "Throw physic to the dogs, he said.
> She did. Next day the dogs were dead."

### A CENSUS OF HALLUCINATIONS.

TO THE EDITOR OF THE HOMŒOPATHIC PHYSICIAN.

DEAR SIR:—May I ask for the publicity of your pages to aid me in procuring co-operation in a scientific investigation for which I am responsible? I refer to the *Census of Hallucinations*, which was begun several years ago by the "Society for Psychical Research," and of which the International Congress of Experimental Psychology at Paris, last summer, assumed the future responsibility, naming a committee in each country to carry on the work.

The object of the inquiry is two-fold: 1st, to get a mass of facts about hallucinations which may serve as a basis for a scientific study of these phenomena; and 2d, to ascertain approximately the *proportion of persons* who have had such experiences. Until the average frequency of hallucinations in the community is known, it can never be decided whether the so-called "veridical" hallucinations (visions or other "warnings" of the death, etc., of people at a distance), which are so frequently reported, are accidental coincidences or something more.

Some eight thousand or more persons in England, France, and the United States have already returned answers to the question, which heads the census sheets. and which runs as follows:

"*Have you ever, when completely awake, had a vivid impression of seeing or being touched by a living being or inanimate object, or of hearing a voice, which impression, so far as you could discover, was not due to any external physical cause?*"

The "Congress" hopes that at its next meeting, in England in 1892, as many as fifty thousand answers may have been collected. It is obvious that for the purely statistical inquiry, *the answer "No" is as important as the answer "Yes."*

I have been appointed to superintend the census in America, and I most earnestly bespeak the co-operation of any among your readers who may be actively interested in the subject. It is clear that very many volunteer canvassers will be needed to secure success. Each census blank contains instructions to the collector, and places for twenty-five names, and special blanks for the "Yes" cases are furnished in addition. I shall be most happy to supply these blanks to any one who will be good enough to make application for them to Yours truly,

(Professor) WM. JAMES.

Harvard University, Cambridge, Mass.

DR. WM. B. CLARKE, Secretary of the Indiana Institute of Homœopathy, and a resident of Indianapolis, is a prolific writer on practical subjects that should more often employ the physician than is common. Articles from the pen of Dr. Clarke in recent issues of *The Sun*, *The Freeman*, and *The Independent*, all published in Indianapolis, on "Care and Dressing of Infants," "The Importance of Pure Air" (this written especially for *The Freeman*, a journal conducted by a colored man for the benefit of colored people), and on "Intra-mural Burial," go to show that followers of Hahnemann give thought to much beside "symptom-hunting."

WHO WAS YOUR GREAT GRANDFATHER.—The *Detroit Journal* desires to receive, by postal card, the address of all living male and female descendants of Revolutionary officers and soldiers of 1776, and when possible, the name, and State of the ancestor. Wonder if W. H. Brearley, proprietor of the *Detroit Journal*, is contemplating a raid upon the national treasury?

OH-DON'T-OLOGY.—*The American Homœopathist* has a page each month containing symptoms of the above disease. There are so many of them accumulated now, that we suggest to the editor that he give us a repertory to them.

### FUN FOR DOCTORS.

"Doctor," said the grateful patient, seizing the physician's hand, "I shall never forget that to you I owe my life." "You exaggerate," said the doctor mildly; "you owe me for fifteen visits; that is the point which I hope you will not fail to remember."

Very sea-sick passenger (feebly)—"O doctor! I'm afraid it's all up with me." Doctor—"Bosh! Nothing up but your breakfast."

"You are accused," said a judge in Paris, "of having attempted to poison your husband with phosphorus. What have you to say?" "I desire that the doctors make an autopsy," replied the woman, as she looked at her husband.—*New Orleans Picayune.*

"I feel sick at heart," said the rejected lover as he leaned upon the railing of the steamer. "I am with you," remarked a fellow-passenger, "only mine is further down."

Female physician—"George, is there any prospect of it clearing off very soon?"

George—"Not much; why?"

Female physician—"Mrs. Smith sent for me to pay her a professional visit three days ago, and I have been waiting ever since for it to clear off. I'm sure she will be expecting me."—*Epoch.*

